# A dual role for glucocorticoid-induced leucine zipper in glucocorticoid function: tumor growth promotion or suppression?

**DOI:** 10.1038/s41419-018-0558-1

**Published:** 2018-04-26

**Authors:** Emira Ayroldi, Lorenza Cannarile, Domenico V. Delfino, Carlo Riccardi

**Affiliations:** Department of Medicine, Section of Pharmacology, Medical School, University of Perugia, Perugia, Italy

## Abstract

Glucocorticoids (GCs), important therapeutic tools to treat inflammatory and immunosuppressive diseases, can also be used as part of cancer therapy. In oncology, GCs are used as anticancer drugs for lymphohematopoietic malignancies, while in solid neoplasms primarily to control the side effects of chemo/radiotherapy treatments. The molecular mechanisms underlying the effects of GCs are numerous and often overlapping, but not all have been elucidated. In normal, cancerous, and inflammatory tissues, the response to GCs differs based on the tissue type. The effects of GCs are dependent on several factors: the tumor type, the GC therapy being used, the expression level of the glucocorticoid receptor (GR), and the presence of any other stimuli such as signals from immune cells and the tumor microenvironment. Therefore, GCs may either promote or suppress tumor growth via different molecular mechanisms. Stress exposure results in dysregulation of the hypothalamic–pituitary–adrenal axis with increased levels of endogenous GCs that promote tumorigenesis, confirming the importance of GCs in tumor growth. Most of the effects of GCs are genomic and mediated by the modulation of GR gene transcription. Moreover, among the GR-induced genes, glucocorticoid-induced leucine zipper (*GILZ*), which was cloned and characterized primarily in our laboratory, mediates many GC anti-inflammatory effects. In this review, we analyzed the possible role for GILZ in the effects GCs have on tumors cells. We also suggest that GILZ, by affecting the immune system, tumor microenvironment, and directly cancer cell biology, has a tumor-promoting function. However, it may also induce apoptosis or decrease the proliferation of cancer cells, thus inhibiting tumor growth. The potential therapeutic implications of GILZ activity on tumor cells are discussed here.

## Facts

- Glucocorticoids (GCs) are crucial therapeutic tools that induce apoptosis in lymphohematopoietic neoplasms.
- In non-hematological malignancies, GCs are used as adjuvant therapy to control the side effects of radiotherapy and chemotherapy, but they can either inhibit or induce tumor growth.
- Most GC-elicited effects result from the transcriptional regulation of GC receptor target genes, such as glucocorticoid-induced leucine zipper (*GILZ*) and its transcriptional variant long-GILZ (L-GILZ).
- GILZ mediates most of anti-inflammatory/immunosuppressive GC effects.
- L-GILZ inhibits tumor growth by p53 activation.

## Open Questions

- Could GILZ mediate GC effects on cancer cell development or inhibition?
- Could GILZ, directly or indirectly, participate in neoplastic cell growth?
- Does L-GILZ have different functions from GILZ in the context of tumor growth?

## Introduction

Glucocorticoids (GCs) are produced by the adrenal gland and regulated by the hypothalamic–pituitary–adrenal axis (HPA). Their secretion follows a circadian rhythm, but it is also controlled by numerous stress stimuli^1^. Natural and synthetic GCs represent the mainstay of therapy for inflammatory and autoimmune diseases, but their role as a cancer therapy is still controversial. Endogenous GCs, together with other stress hormones and neurotransmitters, are key mediators in stress-mediated neoplastic pathologies and significantly affect tumor growth and metastasis^2^.

GCs have both pro- and anti-apoptotic effects. For their proapoptotic properties, GCs are used in lymphohematopoietic cancer^3,4^. In contrast, numerous reports suggest that GCs in non-hematological malignancies, often used as adjuvant therapy, may have tumor-promoting effects^5,6^. Although some therapeutic protocols include GCs (e.g., breast and prostate cancer)^7^, in most tumors their role is still debated and often unknown.

Most GC-elicited effects result from the transcriptional regulation of GC receptor (GR) target genes^8^. Some of the genes upregulated by GCs have been associated to GC sensitivity, such as glucocorticoid-induced leucine zipper (*GILZ, Tsc22d3*). Mouse *Gilz* encodes for a 137-amino acid leucine zipper protein and shares 90% sequence similarity with human *Gilz*^9,10^. Long-GILZ (L-GILZ), a transcript GILZ variant, is upregulated by GCs, but differs from GILZ for some functional features^11^.

GILZ is responsible for most of the anti-inflammatory and immunosuppressive GC effects because it is involved in regulating differentiation, apoptosis, and cell cycle of immune cells^12–14^. GILZ is involved in the modulation of transcription factor activity and signaling pathways implicated not only in immune response but also in inflammation. For example, GILZ associates with and inhibits nuclear factor-κB (NF-κB)-dependent transcription, thus mediating immunosuppressive and anti-inflammatory effects in T cells, macrophages, bone marrow mesenchymal stem cells, synovial endothelium, and bronchial and intestinal epithelium^15–20^. Moreover, GILZ interacts with c-Fos and c-Jun and inhibits activating protein-1 (AP-1)-dependent transcription^21^. It binds Raf^22^ and Ras^23^ and inhibits Ras/Raf downstream target activation, including ERK-1/2 and Akt, thus mediating GC antiproliferative effects^23^. In contrast, L-GILZ binds and inhibits Ras in undifferentiated spermatogonia, contributing to the regulation of spermatogenesis^24^, binds and activates p53 in neoplastic cells, contributing to GC anticancer effects^11,25^, and inhibits the proliferation of thyroid cancer cells^26^.

Thus, GILZ inhibits transcription factors, such as NF-κB^15^ and AP-1^21^, that are implicated in tumor-promoting functions^27,28^ and signaling pathways, such Ras-MEK-ERK, that are involved in pro-tumorigenic effects^29^. L-GILZ activates p53, whose role in preventing tumor development is well established^30^. These observations suggest that GILZ and L-GILZ interfere with the activity of signals important not only in inflammation but also in cancer. However, while much work has been done to understand the immunosuppressive and anti-inflammatory activity of GILZ, little is known of its involvement in cancer development. Here we review the current literature about GILZ and GCs in modulating both transcription pathways deregulated in cancer cells and the immune response during the development of a neoplastic disease. We also discuss the potential biological relevance of GILZ and GCs in controlling cancer growth.

## GCs and cancer

**Fig. 1:**
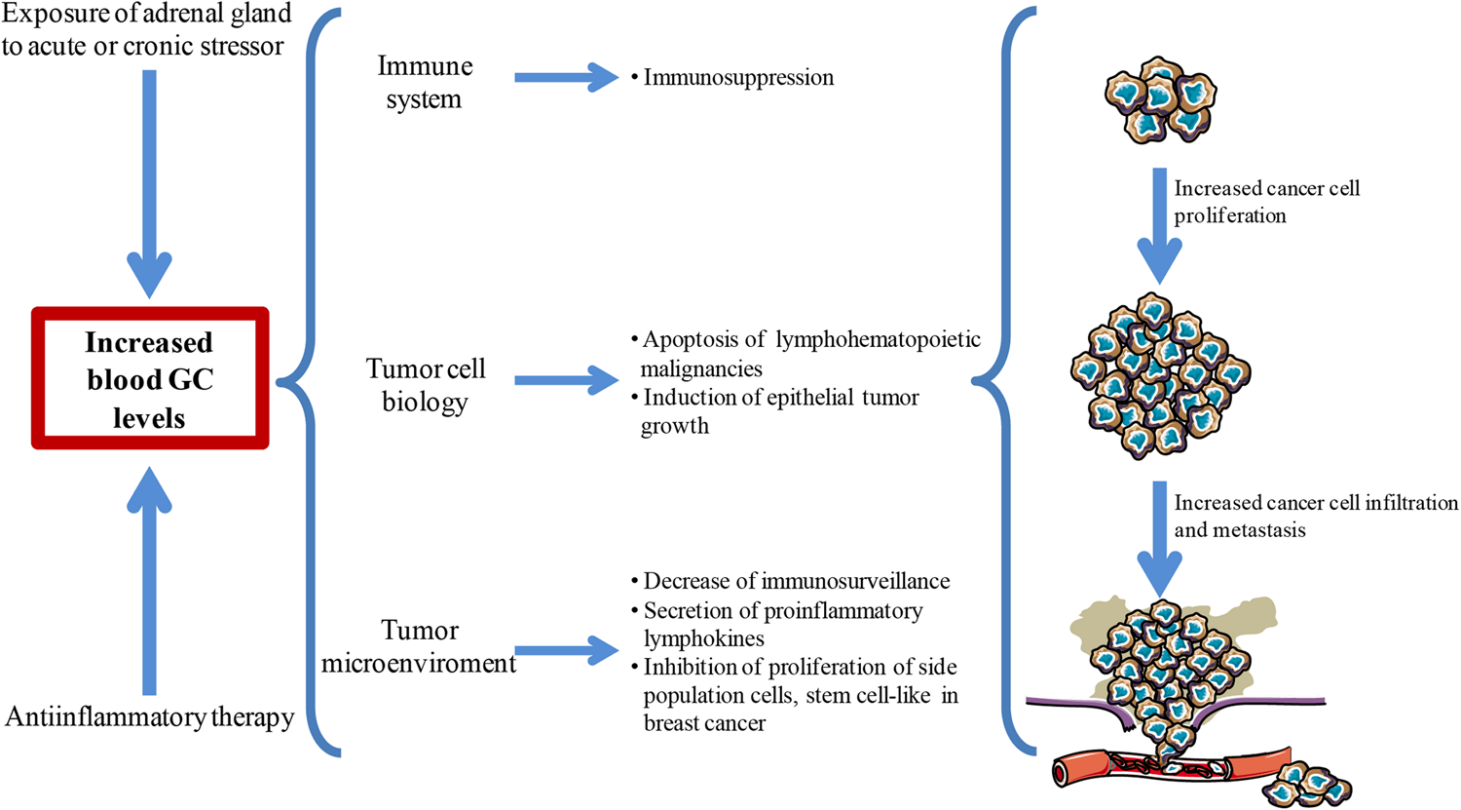
Schematic illustrating the effect of GCs on cancer growth. Increased blood levels of glucocorticoids (GCs), due to either GC anti-inflammatory therapy or acute/chronic stress, could affect tumor growth through activity on multiple levels. GCs can act on all the cells of the immune system, on tumor cells, and on the tumor microenvironment

As mentioned above, GCs are included in several protocols for lymphohematopoietic malignancy treatment^31^ and as a monotherapy or in combination with chemotherapy for solid tumor treatments/adjuvants^32^. However, dysregulation of the HPA with increased GC secretion has been associated with stress- and aging-related cancer^33,34^. Hence, what are the effects on tumor growth when endogenous GCs are increased for stress conditions or when exogenous GCs are administered for inflammatory and non-hematological malignancies diseases? Because the immune system is a main GC target, GCs are involved in stress-induced immune dysregulation, thus affecting cancer. Alteration in GC secretion during chronic stress often results in immunosuppression and a failed immune response against the tumor^2,35^. In many cases, this occurs in patients receiving GC chronic therapy as, if the desired pharmacological effect of exogenous administration of GCs is the control of inflammation and the induction of the immunosuppression, the latter may become dangerous in the presence of a neoplastic disease. Therefore, GCs, by activating GR signaling, may control tumor growth (1) indirectly through modulation of immune system and cells in the tumor microenvironment, or (2) directly by controlling cancer cell survival pathways and affecting tumor cell biology (Fig. 1).

### GCs, immune cells, and cancer

GCs contribute to physiological regulation of the immune system by influencing immune cell cytokine release, apoptosis, and cell proliferation. By inhibiting proliferation and inducing apoptosis, endogenous GCs contribute to physiological development of thymus repertoire and modulation of peripheral T-cell activation^35,36^. The potent regulatory activity of GCs on immune system is also due to the ability of GR to interfere with the activation of many transcription factors that regulate cytokine release, adhesion molecules, receptors, and other factors affected by immune system activity^35^. For example, GR modulates the activity of NF-κB, AP-1^37^, nuclear factor of activated T cells, transducer and activator of transcription (STAT), cAMP response element-binding protein, interferon regulatory factor 3, T-box expressed in T cells (T-Bet), and GATA-3^38,39^.

By acting on one or more of the above transcription factors, GCs affect macrophage and dendritic cell (DC) functions, impairing their antigen-presenting function, decreasing costimulatory and major histocompaibility complex (MHC) class II molecule expression, downregulating proinflammatory cytokines (such as tumor necrosis factor (TNF) and interleukin (IL)-12), and inducing production of anti-inflammatory cytokines, such as IL-10^40,41^. Furthermore, GCs interfere with T-cell activation, inhibit activation-induced cell death, and drive T-helper differentiation^35,42,43^. As a result of these effects, GCs lead to immunosuppression. Under physiological conditions, this immunosuppression is counterbalanced by other factors that regulate the immune system; however, under pharmacological conditions or if chronic stress causes dysregulation of GC secretion, these immunosuppressive effects can enable a tumor to escape immune detection, favoring cancer growth (Fig. 1). This mechanism is so important for tumor survival that cancer cells have “learned” to defend themselves from the immune system through autocrine synthesis of GCs, which is a common feature of malignant cells^44^. For example, colon carcinoma cells synthesize and release GCs, which are implicated in immune system evasion and progression of tumor^45^. Furthermore, dexamethasone (DEX), a synthetic GC, upregulates an inhibitory molecule expressed by activated T cells, programmed cell death 1 (PD-1), and induces T-cell tolerance and tumor growth^46^. This could explain why exogenous and endogenous GCs may negatively influence the cancer response to PD-1-targeting immunotherapy, as highlighted by epidemiological studies^47^.

On the other hand, GC therapeutic activity mimics the physiological effects of endogenous GCs, although with greater potency and efficacy. In other words, they can induce apoptosis of lymphoid cells, which provides grounds for their inclusion in protocols for the treatment of lymphohematopoietic malignancies^31,48–50^ (Fig. 1), such as acute Hodgkin’s lymphoma and non-Hodgkin’s lymphoma, multiple myeloma, lymphoblastic leukemia, and chronic lymphocytic leukemia^51^. The most important molecular mechanisms of GC-triggered apoptosis involve induction of proapoptotic members of the B-cell lymphoma-2 (Bcl-2) family and repression of anti-apoptotic members, such as Bcl-extra-large (Bcl-xL), Bcl-2, myeloid cell leukemia-1 (Mcl-1)^4^. In contrast, the direct effects of GCs on non-hematological cancer cells remain a controversial issue.

### GCs and non-hematological cancer cells

GCs display modest efficacy when used alone or in combination with chemotherapeutic drugs for some non-hematological malignancies, such as breast and prostate cancers^7^. However, in vitro and in vivo studies^32,52^ suggest that exogenously administered GCs, as well as high levels of natural GCs caused by stressor stimuli, may induce epithelial tumor growth (Fig. 1)^7,34^.

In both normal^53^ and cancer cells, the quantity and type of GR isoforms and the affinity of specific GCs for the GRs contribute to the direct effects of GC on tumor biology^2^. Clinical observations suggest that increased GR expression in patients with ovarian cancer correlates with poor prognosis^54^. Increased GR expression may also contribute to progression of castration-resistant prostate cancer^55^ or be responsible for bypassing androgen receptor blockade^56^.

In contrast, other studies suggest that GR functions as a tumor suppressor gene. Matthews et al.^57^ demonstrated that GR promotes chromosome segregation during mitosis and that the GR loss, observed in many cancers, may cause malignant transformation. GR loss or downregulation is also related to epigenetic modifications, such as hypermethylation of its promoter, as observed in a cohort sample of breast cancers^58^. This epigenetic alteration may confer a benefit to cancer cells. In fact, other studies have shown that GR may attenuate estrogen- and androgen-induced proliferation^59,60^, even though GR expression on estrogen receptor-negative breast cancer cells was related to poor prognosis^61^. In small cell lung cancer, GR downregulation correlates with decreased proapoptotic function, an advantage for tumor cell growth^62^.

**Fig. 2:**
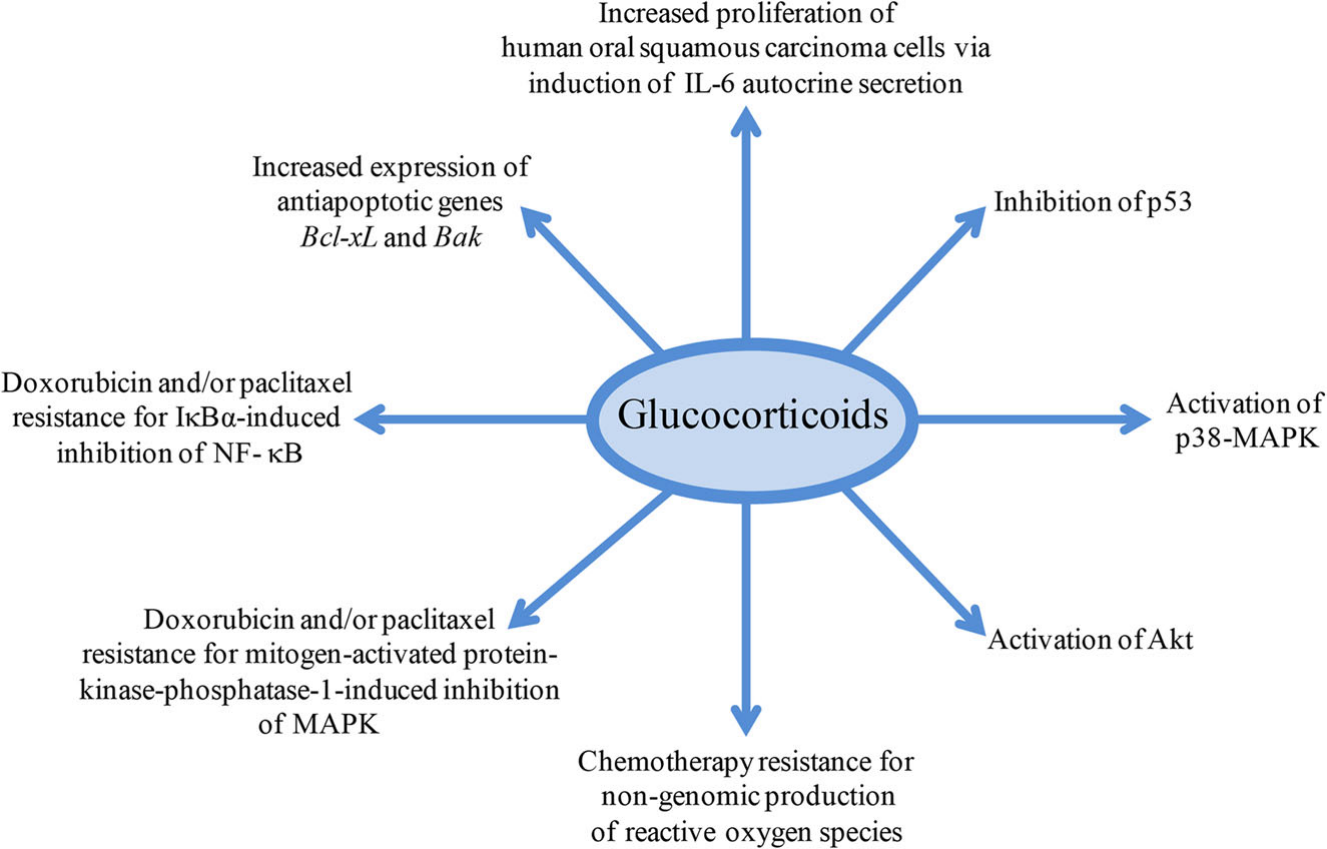
Glucocorticoid and epithelial cancer progression. Glucocorticoid interaction with transcription factors and transduction pathways results in increased proliferation of epithelial cancer cells and resistance to chemotherapy

Many mechanisms underlie the direct effect of GCs on epithelial cancer cell survival. For example, GCs may promote cancer progression by inhibiting the tumor suppressor protein p53^32,63^ or by activating p38-MAPK or Akt, signaling pathways all responsible for GC-induced cell proliferation^6^. In addition, cortisol can stimulate cell proliferation of human oral squamous carcinoma cells via autocrine secretion of IL-6^64^ (Fig. 2).

GCs can induce or protect from apoptosis under different environmental conditions, including the cell type^53^. In fact, GCs may mediate resistance to chemotherapy-induced apoptosis^52,65–67^, by inducing the expression of anti-apoptotic genes. For example, GCs induced the expression of anti-apoptotic genes such as *Bcl-xL* and *Bak*^68^ in a breast cancer cell line. Further, in breast, ovarian, and cervical cancers, GCs inhibit paclitaxel-induced apoptosis through inhibition of NF-κB^69^. Furthermore, anti-apoptotic effects of DEX on breast cancer treated with doxorubicin and/or paclitaxel have been related to DEX-induced mitogen-activated protein kinase (MAPK) phosphatase-1 or IκBα expression, which inhibit MAPK and NF-κB activation, respectively^2^ (Fig. 2). Of note, the inhibition of GC-induced NF-κB and/or MAPK transduction pathways plays a double role: it is critical for the GC anti-inflammatory effects resulting in inhibition of proinflammatory cytokines, but it is also important for GC-mediated cancer cell survival.

Non-genomic, GC-mediated mechanisms may also play a negative role in cancer treatment. As demonstrated in vitro in a breast cancer cell line, GCs interacting with inducible nitric oxide synthase, a key enzyme in generating nitric oxide, increases the production of reactive oxygen species and produces the oxidative damage to DNA that is responsible for a decreased response to chemotherapy^70^ (Fig. 2).

The conflicting observations on the effects of GCs on solid tumors is also due to the number of factors that contribute to modulate the GR response. For example, endogenous or exogenous GCs can affect the tumor microenvironment.

### GCs and tumor microenvironment

The tumor microenvironment plays a key role in tumor growth^71^, and as such, all drug therapies affect both the neoplastic cells and the tumor microenvironment^72^. Hematopoietic cells (T cells, B lymphocytes, natural killer cells, macrophages, DC, and neutrophils), myeloid-derived suppressor cells, mesenchymal cells (stem cells, endothelial cells, fibroblasts, and myofibroblasts), and extracellular matrix are all components of this bidirectional dialogue between tumor and microenvironment cells^73^. For example, tumor-associated stroma has a gene expression profile different from that of normal stroma thus influencing tumor growth^74^. Although many studies have suggested a role of GCs on microenvironment-mediated tumor growth, especially in determining tumor progression, the exact contribution of GR signaling on specific microenvironment cells remains unknown. Nearly all cells in the tumor microenvironment express GR, although to varying degrees^2^. Immune system cells present in the tumor microenvironment are inhibited by GCs, causing a local decrease in immunosurveillance^2^ (Fig. 1). Moreover, tumor-associated fibroblasts, the major components of stromal cells with roles in tumor growth and metastasis, exhibit alterations in GR-induced gene transcription^75^. GR is overexpressed in some stroma breast carcinomas^76^, and increased GC signaling seems to be responsible for insulin resistance in adipocytes, a major component of the breast cancer stroma, with consequent secretion of proinflammatory lymphokines and growth factors implicated in tumor progression^77^. On the other hand, an in vitro experiment has revealed that DEX inhibits the proliferation of a rare cell population, stem cell-like, a side population present in breast cancer stroma (Fig. 1). The result suggests that DEX targets this population and may increase sensitivity of tumor cells to chemotherapy^78^. In conclusion, GCs can slow or promote tumor growth through different mechanisms depending on tumor type, microenvironment, and immune system response. Is GILZ involved in those mechanisms?

## GILZ and cancer

GILZ, through regulation of cell growth and differentiation, immune cell functions, and other molecular mechanisms, mediates most of GC activities, including the immunosuppressive and anti-inflammatory effects^12,13^. Inflammation plays an important role in initiating malignant conversion and promoting tumor growth and metastasis^79^. Inflammatory cells that infiltrate the tumor are an important part of the tumor microenvironment, affecting its growth and aggressiveness. Inflammation, immunomodulation, and cancer initiation and progression occur through common and overlapping pathways^80^. For example, the tumor-promoting functions of NF-κB, STAT3, and AP-1 are correlated to expression of genes that stimulate cancer cell proliferation and survival^81,82^. STAT3 and NF-κB interfere with p53, with a potential tumor-promoting activity^80,83^. It could be hypothesized that GILZ inhibition of NF-κB and AP-1 transcriptional activity^15,21^ may prevent the development of tumor growth via inhibition of proinflammatory cytokines (Fig. 4), despite the fact that DEX-induced NF-κB inhibition may result in unresponsiveness of tumor cells to chemotherapy^2^. In addition, GILZ inhibition of Ras and L-GILZ activation of p53 lead to antioncogenic activity in human cancer^23,30^. However, concomitant GILZ-mediated immunosuppression^13,14^ may favor the development of tumors. Although its function in cancer has not been sufficiently studied, GILZ, much like GCs, may either promote or inhibit tumor growth depending on the context.

### GILZ and immune cells

**Fig. 3:**
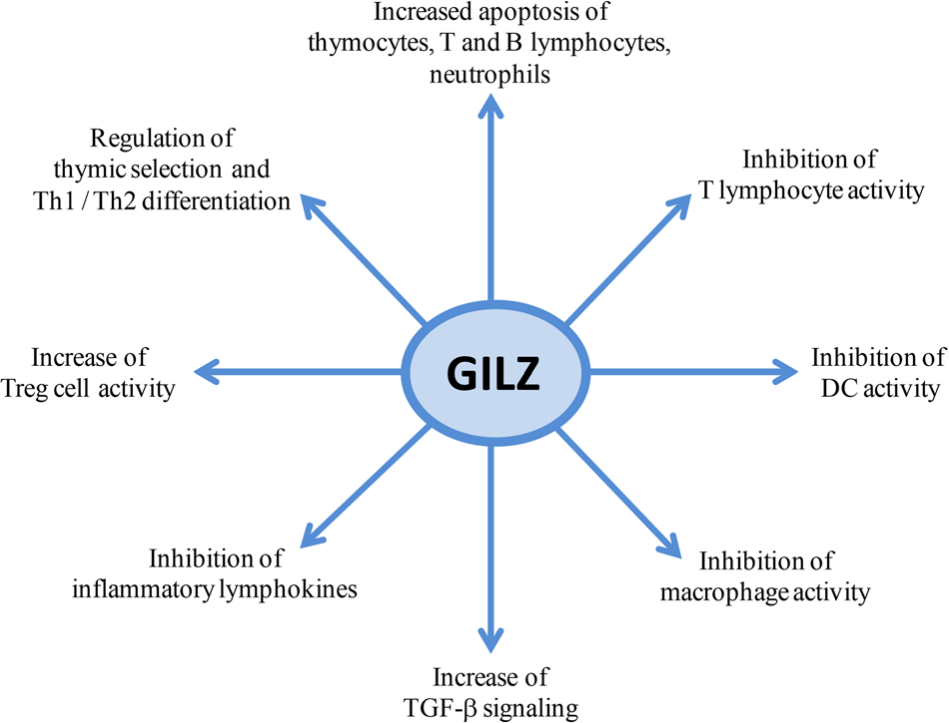
GILZ and immune system. GILZ, through interactions with different transcription factors (as explained in the text), affects the cells of the immune system, thus mediating the physiological and pharmacological effects of GCs. The final outcome of GILZ expression is immunosuppression, which is the desired effect when GCs are administered for therapeutic purposes, but strongly affects tumor growth with an effect that involves both systemic and tumor microenvironment immune cells

GILZ expression negatively modulates immune cell functions^84^ by inhibiting transcription factors crucial for immune system activation^12,13^. Although there is no direct experimental evidence, it may be that GILZ mediates both endogenous stress-associated and exogenous GC effects via its activity on immune cells, causing cancer progression. For example, GILZ, which is upregulated by DEX treatment in mouse and human macrophages, interferes with macrophage and DC functions^85,86^. In DCs, GILZ plays a critical role in the balance between the active and tolerant DC phenotypes, and it is responsible for decreasing expression of costimulatory and MHC class II molecules and for the generation of T-regulatory (Treg) cells^87^. Moreover, antigen-specific, IL-10-producing Treg cells, generated by GILZ-expressing DCs, inhibit the T-lymphocyte response^88^ (Fig. 3). Furthermore, GILZ increases Treg cell activity by favoring transforming growth factor β (TGF-β) signaling^89^. In T lymphocytes, GILZ regulates T-lymphocyte differentiation, activation, and apoptosis through mechanisms involving thymic selection and Th-1 and Th-2 differentiation^90,91^. The role of GILZ as an immunosuppressive molecule has been validated by transgenic mouse models (GILZ-TG). For example, mice with forced GILZ expression in DC^87^ or in bone marrow-derived mesenchymal stem cells (BMSCs)^92^ exhibit an accumulation of Tregs. Furthermore, GILZ-TG mice, in which GILZ is selectively overexpressed in T cells, exhibit NF-κB inhibition and consequently lower T-cell activation; however, these mice are also less susceptible to experimental colitis^18^ or spinal cord injury^93^, indicating that GILZ similar to GCs has both immunosuppressive and anti-inflammatory activities.

In addition, mice lacking GILZ showed accumulation of B precursors in the bone marrow and B lymphocytes in peripheral lymphoid organs^94^, suggesting a role for GILZ in B-cell apoptosis. Moreover, GILZ led to apoptosis in human neutrophils via downregulation of an anti-apoptotic protein, Mcl-1^95^. The final outcome of GILZ effect on the immune system is immunosuppression that promotes tumor growth (Fig. 3).

**Fig. 4:**
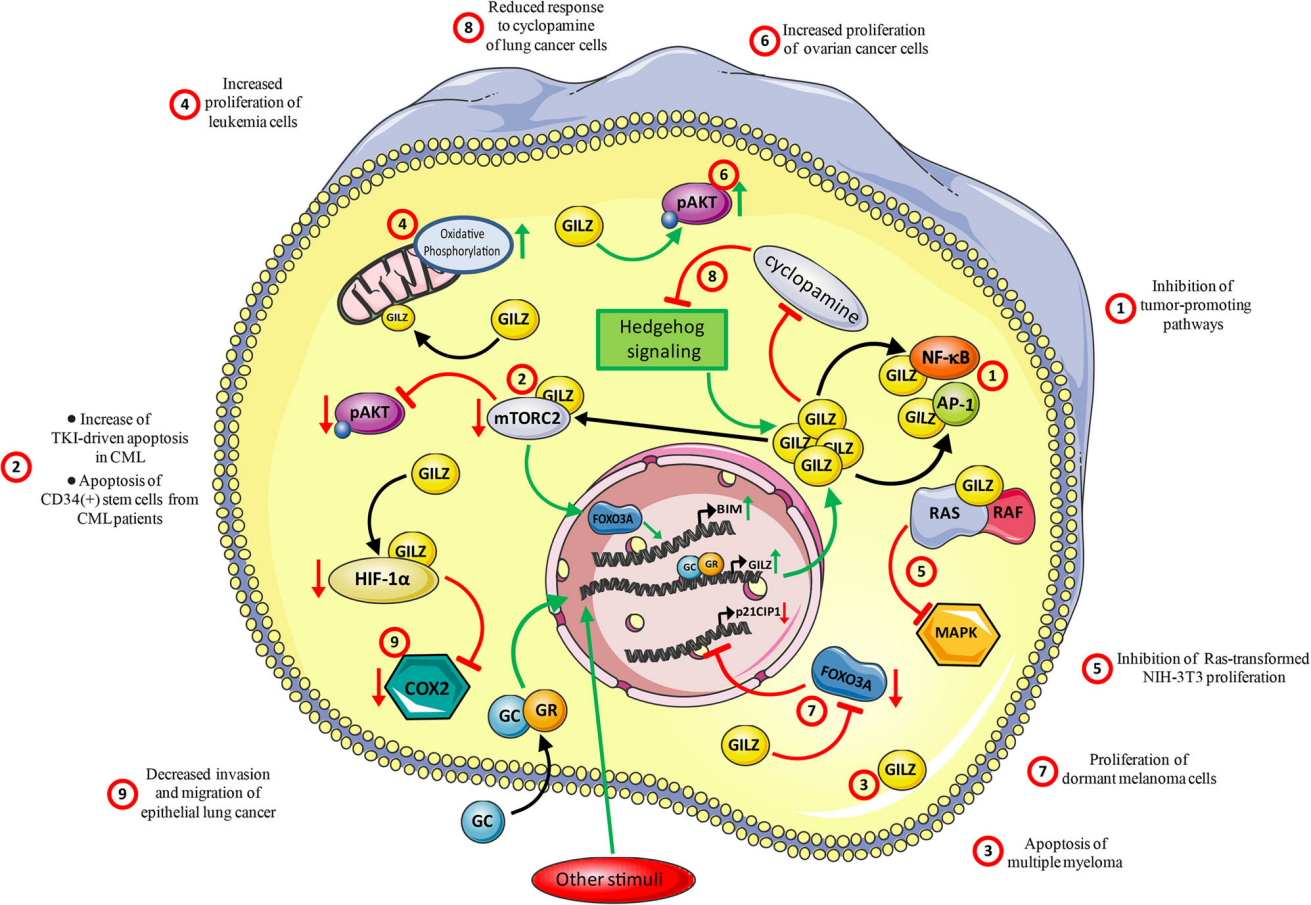
GILZ and cancer cells. Through its interaction with different molecules and/or pathways, GILZ has contrasting effects on cancer cells. By binding and inhibiting NF-κB and AP-1, GILZ may inhibit tumor-promoting factors (1); by binding and inhibiting the mammalian target of rapamycin complex-2 (mTORC2) and activating FOXO3Α-mediated transcription of the proapoptotic protein, Bim, GILZ increases the apoptosis induced by tyrosine kinase inhibitors (TKI) in chronic myeloid leukemia (CML) and increases apoptosis of CD34^+^ stem cells isolated from CML patients (2). In myeloma cells, through an unknown mechanism, GILZ mediates GC apoptotic effects (3). GILZ inhibits the proliferation of Ras-transformed-NIH-3T3 cells (through inhibition of the Ras pathway) (5). GILZ induction increased proliferation of leukemia cells (by increasing mitochondrial oxidative phosphorylation) (4), ovarian cancer cells (by activation of pAkt) (6), and murine dormant melanoma cells (via the inhibition of FOXO3A and its downstream target, the cyclin-dependent kinase inhibitor 1 [p21CIP1]) (7). In lung tumor cells, the data are conflicting: GILZ reduces the response to cyclopamine by affecting Hedgehog signaling, which is responsible for GILZ expression (8), but decreases the migration and invasion of lung cancer cells by binding and targeting hypoxia-responsive transcription factor-1α (HIF-1α) for proteasome degradation, thereby decreasing COX-2 expression (9). Red T-headed leaders indicate inhibition; green arrow-headed leaders indicate activation

The effect of GILZ resembles that of GCs in its physiological control of apoptosis. In fact, thymocytes from GILZ-TG mice activate caspase-8, downregulate Bcl-xL, and undergo apoptosis, suggesting that GILZ, much like GCs^50^, regulates the thymic repertoire^90,96,97^. However, GILZ may also protect from apoptosis. GILZ overexpression rescues both the CTLL-2 cell line from IL-2 withdrawal-induced apoptosis by inhibiting Bim expression^98^ and mature T lymphocytes from T-cell receptor-induced apoptosis by inhibition of NF-κB activation^9^. This last phenomenon resembles the GC-like mechanism of mutual exclusion^42^. In addition, GILZ expression has been described in myeloma^99^, lymphoblastic leukemia, and chronic myeloid leukemia (CML)^100^, suggesting that GILZ has a role in the pharmacological GC-mediated apoptosis of myelodysplastic disorders. In CML, GC-induced GILZ expression abolished resistance to tyrosine kinase inhibitors, a drug family used in this syndrome, by inactivating the mammalian target of rapamycin complex-2/AKT signaling pathway and by activating FOXO3A-mediated transcription of the proapoptotic protein Bim, which plays a critical role in cell death induced by tyrosine kinase inhibitors^100^. By the same mechanism, GILZ may contribute to apoptosis of CD34^+^ stem cells isolated from CML patients^100^. In multiple myeloma, for which GCs are commonly used as effective therapeutics, decreasing GILZ levels by siRNA knockdown inhibited GC-induced cell death^99^ (Fig. 4). Accordingly, only a subset of myeloma patients responds to high-dose DEX: those showing transcriptional activity of GR and GILZ expression, whose silencing, again, reverts DEX-induced apoptosis^101^.

In contrast, recent studies have shown that GILZ reprograms the metabolism of the neoplastic cell, which is essentially based on glycolysis, increasing its mitochondrial oxidative phosphorylation, thus giving to leukemic cells a proliferative, metabolic advantage^102^ (Fig. 4). The increase of mitochondrial oxidative metabolism has been demonstrated in vitro in both cells overexpressing exogenous GILZ and DEX-treated cells overexpressing endogenous GILZ. The increased proliferation of cells overexpressing GILZ is counterbalanced by an augmented susceptibility to the cytotoxic effects of pro-oxidative, mitochondria-specific drugs (e.g., elesclomol). However, this mechanism is observed in cells expressing exogenous GILZ, but not in those treated with DEX, suggesting that DEX interferes with mitochondrial oxidative phosphorylation-mediated cell death in a GILZ-independent manner^102^. In addition, the increased mitochondrial oxidative phosphorylation may explain how GILZ protects cells from endoplasmic reticulum (ER) stress-mediated apoptosis, which is a consequence of genetic and epigenetic aberration in many types of cancer^103^, contributing to cancer cell survival under ER stress. In this case, cells overexpressing exogenous GILZ behave as those expressing DEX-induced endogenous GILZ, suggesting that GILZ mediates the effects of GCs^103^. Accordingly, endogenous GCs protect from ER stress in the intestinal epithelium, helping to maintain homeostasis and promoting drug effects in cases of inflammatory bowel disease^104^. Although an important limitation of many of these studies is that they are performed exclusively in vitro, they confirm that the effects of GILZ, much like those of GCs, depend on cell type, environmental conditions, and concomitant stimuli, producing different outcomes.

### GILZ and epithelial cancer cells

Constitutive activation of the MAPK signaling pathway, which regulates physiological proliferative events, is often found in inflammation, driving proinflammatory cytokine expression and often contributing to cancer initiation or development^29^. An interesting involvement of GILZ in GC anti-inflammatory and immunosuppressive effects, which may also impact with cancer, is its ability to inhibit MAPK pathways through binding to some components of the MAPK cascade^105^. Indeed, GILZ interacts with Ras and Raf, leading to inhibition of downstream Ras-dependent signaling pathways^106^. By this mechanism, GILZ can mediate the DEX antiproliferative effect on activated T lymphocytes and exert antioncogenic activity in vivo and in vitro in Ras-transformed NIH-3T3 cells^106^ (Fig. 4). Many epithelial tumors are characterized by the hyperactivating mutations found in Ras or BRAF^107^. One completely unexplored field involves GILZ effect in human cancer. It is plausive that GILZ may have antioncogenic activity by, for example, binding to Ras and/or Raf. Instead, GILZ expression was observed in the cytoplasm of cancer cells, but not in the epithelium of normal ovaries and benign tumors, suggesting its potential importance for the proliferation of ovarian cancer cells^108^. In addition, an in vitro model with a cell line derived from ovarian cancer suggested that GILZ enhanced phosphorylated Akt expression and activity, leading to increased proliferation^108^ (Fig. 4). Therefore, GILZ^109^ and GR^54^ expression correlates with poor prognosis in ovarian cancer. In contrast, in estrogen-dependent breast cancer, estrogens downregulate GILZ expression, although the functional meaning of this discovery remains undetermined^110^.

The role of GILZ has been studied in dormant cells of murine melanoma^111^. These are cells in mitotic reversible quiescence, present in cancer-treated and healthy individuals alike. Their activity is regulated by dormancy-associated genes and microenvironment stimuli^112^. GILZ signaling, via the inhibition of FOXO3A and its downstream target, the cell cycle inhibitor, cyclin-dependent kinase inhibitor 1 (p21CIP1), antagonizes cell quiescence, induces cell cycle reactivation and tumor development in dormant murine melanoma cells. Furthermore, GILZ repression induces cellular quiescence, contributing to melanoma inactivity^111^ (Fig. 4).

High GILZ levels have also been associated in lung cancers with a reduced response to cyclopamine, a drug that inhibits Hedgehog signaling, which induces GILZ^113^, but is often deregulated in many tumors^114^. In contrast, it has been demonstrated that GILZ by a mechanism involving the inhibition hypoxia-responsive transcription factor-1α (HIF-1α) decreases invasiveness of epithelial lung cancer cells^115^. Aerobic glycolysis is a peculiar characteristic of the neoplastic cell. In hypovascularized tumor areas, hypoxia induces HIF-1α production, which intensifies anaerobic glycolysis and lactate production, suppresses the immune response, and induces invasion and metastasis. According to some studies, hypoxia causes downregulation of GR and thereby reduces the effectiveness of GCs^116^, while other studies suggest that hypoxia causes GR upregulation and increased sensitivity to GCs^117^. A recent study proposes that GCs, via GILZ expression, suppress hypoxia-induced cyclo-oxygenase of type 2 and HIF-1α activity and decrease migration and invasion of certain epithelial lung cancer cell lines^115^ (Fig. 4).

In summary, GILZ expression appears to have opposing effects on the neoplastic cell metabolism. It can (1) reprogram the neoplastic cell toward oxidative mitochondrial phosphorylation, as seen before with leukemic cell lines, conferring to tumor cells a proliferative advantage^102^, or (2) inhibit HIF-1α and decreases its signaling pathway, weakening anaerobic metabolism in lung neoplastic cells, thus hindering cancer invasiveness^115^. Although some of these observations have limits related to the model (exclusively in vitro), once again the effect of GILZ, just like GCs, seems to be related to the cellular type and presence of other stimuli, such as those from microenvironment.

### GILZ and tumor microenvironment

GILZ has not been studied thoroughly in the tumor microenvironment, but it is a crucial mediator of GC-immunosuppressive effects. Its role in the dialogue between the tumor and the microenvironment cells is highly plausible. GILZ could, in fact, influence all the cells of the immune system that infiltrate the tumor microenvironment, as it does with the cells of the systemic immune system^13,14,118^. As mentioned above, GC-treated DCs express GILZ, responsible for their tolerant phenotype^87,119^. One recent study demonstrates in vivo and in vitro that, as a result of tumor/microenvironment interaction, GILZ is highly upregulated in tumor microenvironment DCs, resulting in the suppression of immune T-cell response against cancer, which can be restored via GILZ blockade^120^. Thus, in the tumor microenvironment, where GILZ could be upregulated in macrophages or DCs by immunosuppressive lymphokines such as IL-10^16^, the tolerogenic antigen-presenting cell phenotype may induce not only tumor-specific T-cell inhibition but also Treg cell activity. As described above, GILZ increases the TGF-β signal that not only leads to Treg cells augmentation^89^ but also plays an important role in tumor development, both positively and negatively, for example, by its ability to control inflammatory/immune cell and fibroblast infiltration into the tumor microenvironment^121^.

Other indirect evidence suggests a role of GILZ in the tumor microenvironment. BMSCs, engineered for stable GILZ expression, inhibit lymphocyte proliferation in a mixed lymphocyte reaction assay. Furthermore, GILZ expressed in BMSCs increases the production of IL-10 and decreases the production of IL-6, resulting again in an increase of Treg cell production and activity^92^. On the other hand, GILZ antagonizes the inhibitory effect of TNF-α on marrow mesenchymal stem cell osteogenic differentiation via the inhibition of TNF-α-induced ERK activation^122^.

As already seen, GILZ is involved in controlling the cellular cycle of dormant melanoma cells, especially those with stem cell characteristics^111^. One can hypothesis that signal exchanges between different cells in microenvironment may affect GILZ expression in dormant cells, with the end result being promotion or hindrance of cancer cell growth.

## L-GILZ and cancer

L-GILZ is a transcriptional variant of the well-established and studied GILZ^11^. L-GILZ is upregulated by GCs, albeit at different levels and in different organs and tissues than GILZ. Due to its interaction with several different signaling proteins, L-GILZ can affect multiple outcomes. L-GILZ regulates spermatogenesis through interacting and inhibiting Ras^24^. L-GILZ controls myogenesis by binding and inhibiting MyoD, a crucial factor for muscle cell differentiation that regulates MyoD/HDAC1 transcriptional activity, thus mediating the anti-myogenic effect of GCs^11^. In addition, L-GILZ activates p53, and through that mechanism activates p53-dependent antiproliferative and proapoptotic pathways, resulting in inhibited tumor cell growth^25,30^.

L-GILZ’s antioncogenic activity has been demonstrated particularly in overexpression models. Indeed, L-GILZ forced expression in p53^+/+^, but not in p53^−^^/−^, HCT116 human colorectal carcinoma cells reduces cell proliferation and suppresses the growth of xenografts in mice^30^. The underlying molecular mechanism involves the ability of L-GILZ to dissociate the MDM2/p53 complex and inhibit MDM2-mediated proteasome degradation of p53, resulting in p53 activation and transcription of downstream genes *p21* and *PUMA*^30^. Further strengthening the biological significance of these observations, L-GILZ was shown to mediate antiproliferative DEX activity by this mechanism. Indeed, L-GILZ, upregulated by DEX in MCF7 breast cancer cells, binds and activates p53 and induces activation of PUMA and p21, inhibiting cell proliferation. This effect is essentially due to L-GILZ as its silencing reverses the antiproliferative activity of DEX^30^.

Recently, L-GILZ has been shown to play a role in the proliferation of thyroid cancer cells. L-GILZ is expressed in highly differentiated thyroid cancer cells, while it is absent in less-differentiated or anaplastic cells^26^. Moreover, its functional role in thyroid cancer is derived from the observation that its overexpression in L-GILZ-deficient cells significantly inhibits their proliferation. In addition, L-GILZ is involved in the antiproliferative effect of kinase inhibitor drugs targeting the MAPK kinase signaling pathway, which is often constitutively activated in both the thyroid and other cancers^29^. Pharmacologically treating thyroid cancer cells carrying a BRAF mutation (which hyperactivates the MAPK pathway) with kinase inhibitors upregulates  L-GILZ and inhibits cellular proliferation. Notably, L-GILZ silencing reverses the antiproliferative activity of the MAPK inhibitors. Moreover, a fusion protein containing L-GILZ, injected into mice transplanted with thyroid cancer cells was found to reduce the growth of xenografts^26^^.^

These are initial observations that generate many new questions. For example, could L-GILZ be essential for GC/p53 cross talk and, by this newly described mechanism, contribute to GC therapeutic efficacy? Could the upregulation of L-GILZ, induced by MAPK inhibitor drugs, and its functional significance be a phenomenon involved in multiple tumors and multiple antiproliferative drug mechanisms? And finally, could L-GILZ become an antitumor drug? Several experimental approaches could be taken to answer these intriguing questions.

## CONCLUSIONS

In this review, we discussed the possible involvement of GILZ (and L-GILZ) in the effect GCs have on tumor growth. While the role of GILZ as a key mediator of anti-inflammatory and immunosuppressive GC effects is widely accepted and documented, its ability to mediate tumor cell growth inhibition or promotion has only been hypothesized. In fact, the attention of researchers has primarily been focused on the anti-inflammatory activity of GILZ, with the aim of obtaining a GILZ drug with fewer side effects than GCs, which could replace GCs. Consequently, until now, the role of GILZ on the other physiological and pharmacological effects of GCs, including its activity on cancer, remains poorly understood.

*GILZ*, as an immunosuppressive gene, participates in GC-mediated immunosuppressive effects on both systemic and tumor microenvironment immune cells and thus, by this mechanism, may promote tumor growth. Meanwhile, GILZ is responsible for GC-driven apoptosis in lymphohematopoietic cells, but promotes the growth of ovarian cancer cells via direct effect on tumor cells. Like GCs, GILZ can either favor or inhibit tumor development, depending on the context. Nevertheless, the ability of GILZ and L-GILZ to inhibit Ras, and that of L-GILZ to activate p53, shows their ability to modulate pathways and signals important for the development and progression of the neoplastic cell. Thus, is it plausible that GILZ and L-GILZ are involved in neoplastic pathologies regardless of their ability to mediate the effects of GCs. This observation could develop new field for investigation.

With the finding that GILZ controls the proliferation of dormant melanoma cells, especially those with stem cell characteristics, an intriguing new field of investigation has opened up that may link GCs, GILZ, the immune system, and neoplastic development. Although a role for GILZ has not been studied in the dormant cells of other malignancies, GILZ could play a general regulatory function in tumor dormancy. If this hypothesis is true, future exploration could include the signals regulating GILZ expression and function. In fact, numerous and overlapping stimuli from the microenvironment could control GILZ expression in dormant cells, either directly or indirectly through the involvement of immune cells. Moreover, stress-induced or exogenously administered GCs could act by regulating GILZ expression in dormant cells. This would confer to GILZ a decisive role in the fate of a neoplastic cell. Consequently, the pharmacological modulation of the tumor microenvironment, which may also involve GC and GILZ, becomes a critical potential therapeutic intervention.
